# The Effect of Cognitive Behavioral Therapy on Depression, Anxiety, and Stress in Patients With COVID-19: A Randomized Controlled Trial

**DOI:** 10.3389/fpsyt.2020.580827

**Published:** 2020-10-30

**Authors:** Jinzhi Li, Xiuchuan Li, Jie Jiang, Xuexue Xu, Jing Wu, Yuanyuan Xu, Xin Lin, John Hall, Huashan Xu, Jincheng Xu, Xiaoyue Xu

**Affiliations:** ^1^Department of Nursing, Bengbu Medical College, Bengbu, China; ^2^Department of Nursing, The First Affiliated Hospital of Bengbu Medical College, Bengbu, China; ^3^Department of Stomatology, The First Affiliated Hospital of Bengbu Medical College, Bengbu, China; ^4^Department of Respiratory, The First Affiliated Hospital of Bengbu Medical College, Bengbu, China; ^5^Department of Intensive Care Unit, The Third People's Hospital of Bengbu, Bengbu, China; ^6^School of Population Health, University of New South Wales, Kensington, NSW, Australia; ^7^Department of Psychiatry, Bengbu Medical College, Bengbu, China

**Keywords:** cognitive behavioral therapy (CBT), COVID-19, DASS-21, psychological health, depression, anxiety, stress

## Abstract

**Background:** As a public health emergency of international concern, the COVID-19 outbreak has had a tremendous impact on patients' psychological health. However, studies on psychological interventions in patients with COVID-19 are relatively rare.

**Objectives:** This study examined the effectiveness of Cognitive Behavioral Therapy (CBT) in relieving patients' psychological distress during the COVID-19 epidemic.

**Methods:** Ninety-three eligible participants selected by cluster sampling were randomized to an intervention group (*N* = 47) and a control group (*N* = 46). Participants in the control group received routine treatment according to the Chinese Management Guidelines for COVID-19, while participants in the intervention group received routine treatment with additional CBT. The Chinese Version of Depression Anxiety and Stress Scale-21 (DASS-21) was used to evaluate depression, anxiety, and stress for all participants at baseline and post-intervention. Two-sided *t-*test, and proportion tests were used to examine the differences between the intervention and control group for each DASS-21 indicator. Univariate linear regression was used to examine the association between chronic disease status and change in each DASS-21 indicator after intervention. Two-way scatter plots were generated to show the association of the length of hospital stay and the changes of each DASS-21 indicator by intervention and control groups.

**Results:** Significant decreases in means were found for scales of depression, anxiety, stress and total DASS-21 in both intervention (*p* < 0.001) and control group (*p* = 0.001), with participants in the intervention group having a bigger reduction in means. After the intervention, more participants in the intervention group had no depression or anxiety symptoms than in the control group, but no statistical differences were found (*p* > 0.05). Compared with participants with chronic disease, participants with no chronic disease had a significantly larger reduction of total DASS-21 scale (coefficient = −4.74, 95% CI: −9.31; −0.17).The length of hospital stay was significantly associated with a greater increase in anxiety scale in the intervention group (*p* = 0.005), whilst no significant association was found in the control group (*p* = 0.29).

**Conclusions:** The patients with COVID-19 experienced high levels of anxiety, depression and stress. Our study result highlights the effectiveness of CBT in improving the psychological health among patients with COVID-19, also suggests that CBT should be focused on patients with chronic disease and those who have longer hospital stays. These results have important implications in clinical practice in improving psychological health in the context of COVID-19 pandemic.

**Trial Registration:** ISRCTN68675756. Available at: http://www.isrctn.com/ISRCTN68675756.

## Introduction

Coronavirus disease-2019 (COVID-19) is a respiratory infectious illness caused by a new virus. In 30 January 2020, The World Health Organization declared the COVID-19 outbreak a public health emergency of international concern (1). By 6 June 2020, COVID-19 had affected more than 7 million people worldwide in 213 countries, resulting in more than 400,857 deaths (2). COVID-19 has resulted in a significant burden on health systems as well economic development, along with a significant impact on individual's physical, and psychological health.

In China, during the initial stage of the COVID-19 outbreak, the number of infected and confirmed cases increased rapidly in a short period due to a lack of knowledge of this new infectious disease. The data shows that on January 27, 2020, there were 4,515 cases confirmed in mainland China, of which 76.0% were patients with mild symptoms (3). Later, with the improvement of COVID-19 nucleic acid detection tests, the number of diagnosed patients increased dramatically to 14,380 by February 1, 2020, and the proportion of patients with mild symptoms increasing to 83.2% (4). Given the significant concerns around the spread of COVID-19, many people were under extreme psychological stress, with an increased risk of panic, fear, anxiety and depression (5–8).

Current studies on the incidence of psychological distress in patients with COVID-19 vary significantly depending on the differences in study setting, location and sample size. Kong et al. (9) investigated 144 patients in a Wuhan hospital using the Hospital Anxiety and Depression Scale and found that 28.5–34.7% of participants had symptoms of anxiety and depression, of which more than half had moderate to severe symptoms of anxiety or depression. Cheng et al. (10) used the Anxiety Self-rating Scale to investigate 76 patients in a Hangzhou hospital and found that the incidence of anxiety was 65.8%, of which 22.4% had moderate to severe anxiety symptoms. A cross-sectional study (11) from a Wuhan hospital showed that among 60 participants, the incidence of anxiety, tension and depression were 47.5, 64.3, and 27.1%, respectively. The main causes of psychological stress that identified from previous studies include: (1) it always takes a long time for the patients to be diagnosed with COVID-19, (2) always received misleading information from the media, friends or colleagues, such as wrong disease statistics and disease management strategy, which may cause confusion and panic (6, 12), (3) worried about the uncertainty of the treatment effect (13), (4) side effects of treatment such as insomnia and nausea that can aggravate mental distress, (5) worried about the families be infected because of their close contacts (14), and (6) the isolated medical environment which patients have never been experienced in their life. To help patients with COVID-19 release their psychological stress, it is urgent to provide psychological intervention in the clinical practice.

Previous studies have reported that during the early stage of public health outbreak emergencies (e.g., SARS), people suffer from anxiety, depression and psychotic symptoms, and these may lead to extreme outcomes such as suicide (15, 16). The existing research also highlights that patients with COVID-19 suffer high levels of anxiety, depression, loneliness, despair and anger (17). Results from a meta-analysis (12) showed that the percentage of anxiety and depression symptoms in patients with COVID-19 was significantly higher than that of the public and front-line medical staff. A small number of patients demonstrate extreme psychological behaviors during COVID-19 pandemic, such as blaming, abusing medical staff and tearing up protective equipment, which exposed front-line medical staff to a higher risk (18).

In addition, multiple studies have documented that without timely psychological intervention and assistance, psychological symptoms such as anxiety and depression can further develop into severe mental disorders such as acute stress disorder (ASD) and post-traumatic stress disorder (PTSD). The psychological disorders on patients resulting from the COVID-19 outbreak have been widely reported (9–12, 17, 19, 20). For example, Wu et al. (19) used the post-traumatic stress disorder checklist (PCL-5) to assess PTSD and found among 8 patients with COVID-19 (2 confirmed cases and 6 suspected cases), one confirmed case and one suspected case were diagnosed with PTSD. One case study reported that 2 patients with COVID-19 developed ASD during quarantine (20). These psychological disorders impact on patients' quality of life and further increased the psychological and economic burden for their family members (21–23). Therefore, effective psychological intervention at the early stages of COVID-19 is important for patients.

Cognitive behavioral therapy (CBT) as an evidence-based psychotherapy has been widely used in the treatment and prevention of physical and psychological distress in both the community (24, 25) and inpatients (26, 27). CBT is a series of methods, including cognitive reconstruction, behavioral change and social support, with aims to help individuals to identify stress levels and modify negative cognitive beliefs and behaviors (28), reduce or eliminate symptoms of psychological distress, and further help individuals back to their normal life in terms of psychological and social functions. Many randomized controlled trials (RCT) have highlighted that participants who received CBT had a significant reduction in anxiety and depression levels improving their quality of life. It was found that even a short program of CBT can improve patients' psychological stress and somatic symptoms such as insomnia (29). Results from a meta-analysis has shown studies have reached a consents that CBT is the most effective and economical psychotherapy to relieve psychological distress and related physical symptoms, such as insomnia and physical fatigue (30, 31).

Despite these widely reported studies on the psychological health of patients with COVID-19, most studies are limited to a cross-sectional design. In addition, to the best of our knowledge, there is no study in China that has conducted an RCT investigating a CBT intervention to patients with COVID-19, with the aim of improving their psychological health. The aim of this study was to apply CBT to patients with COVID-19 and examine the effectiveness of CBT in relieving patients' psychological distress during the COVID-19 epidemic.

## Methods

### Study Design and Setting

This RCT was conducted in the First Affiliated Hospital of Bengbu Medical College located in Bengbu, Anhui Province. This has been a designated COVID-19 treatment hospital throughout the epidemic. The intervention was delivered by nurses who have received professional training in CBT as well as having COVID-19 prevention and treatment knowledge and treatment training.

### Participants

All participants were collected using cluster sampling from the First Affiliated Hospital of Bengbu Medical College. All participants expressed their willingness to participate in the study during the period from February 4 to March 3, 2020. The range of hospital admission were from 7 to 29 days.

A total of 109 patients were included at the initial screening. The inclusion criteria were: (1) positive for the COVID-19 by the real-time fluorescent reverse transcription polymerase chain reaction (RT-PCR) test for the detection of nucleic acid in respiratory specimens with throat swabs and sputum specimens, (2) patients with COVID-19 who had mild symptoms in line with the diagnostic criteria of the Chinese Management Guidelines for COVID-19 (32), and (3) had good communication and understanding of Chinese. The exclusion criteria were: (1) previously diagnosed with depression and currently taking medication, (2) had prior cognitive dysfunction and (3) had experienced another major stressful event (e.g., divorce, bereavement) in the past year.

**Figure 1:**
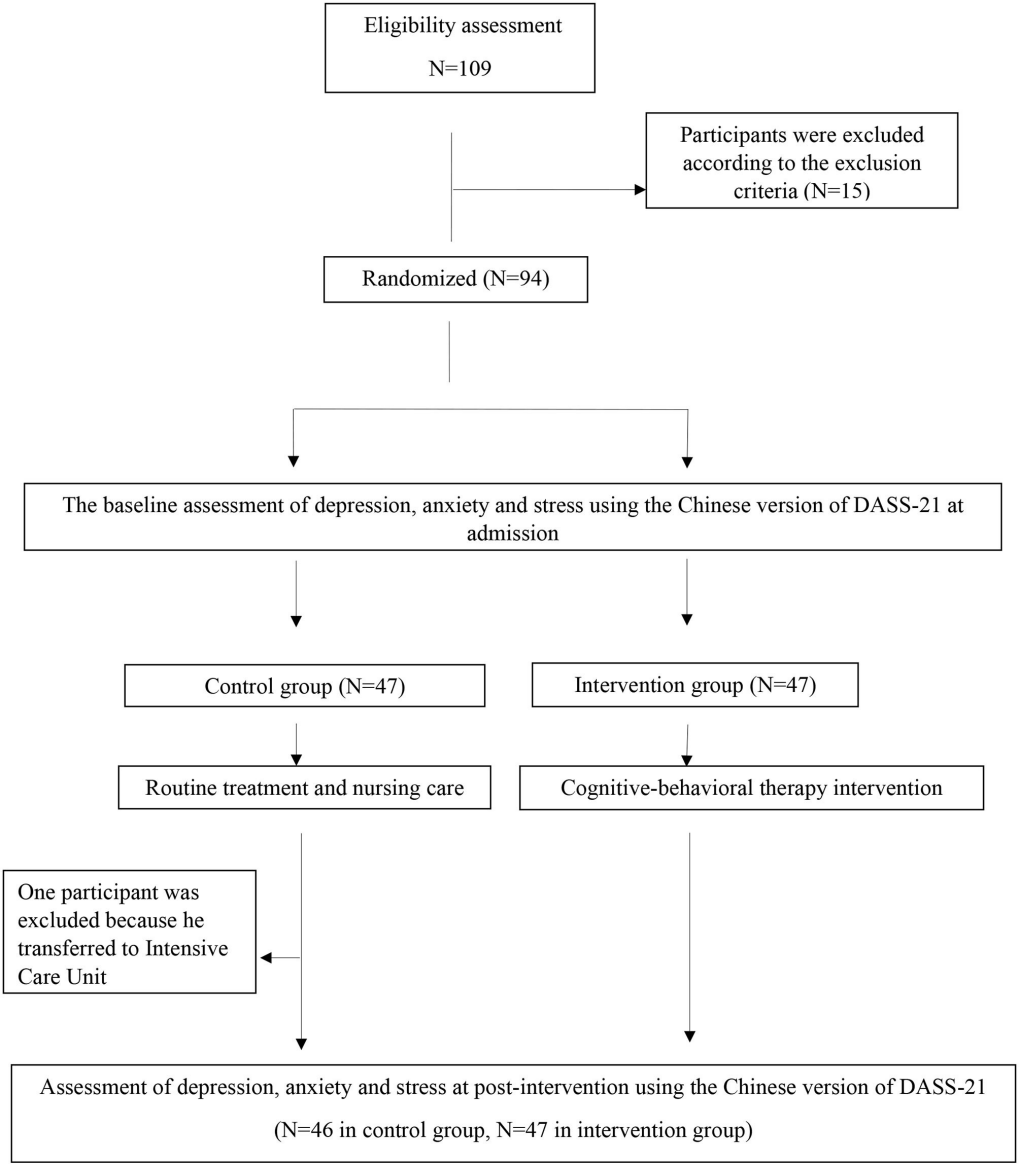
Flow chart of the study procedure.

Based on the exclusion criteria 15 patients were excluded. The sample consists of 94 participants who were selected and randomly divided into the intervention group (*N* = 47) and the control group (*N* = 47) using a computerized random number generator by a trial statistician who had no clinical involvement in the project. Furthermore, participants who were transferred to Intensive Care Unit (ICU) for further treatment were excluded (*N* = 1, in the control group) as disease progression did not permit participation in the entire study. Study participants in the control group received routine treatment (32), while participants in the intervention group received the routine treatment with the additional of CBT. The final sample was made up of 93 participants, with 47 in the intervention group and 46 in the control group. The study procedure is shown in Figure 1.

The sample size was consistent with Lancaster et al. (33) study which suggested that 30 or more patients are enough for a pilot study. This gives adequate power to detect a difference between two groups. This study was approved by the Ethics Committee of the First Affiliated Hospital of Bengbu Medical College. All participants were informed of: (1) the purpose of the study, (2) their ability to withdraw from the study at any time, and (3) that the study findings would be written up as a peer-reviewed publication. All participants provided signed informed consent to participate in the study.

### Measurements

The demographic characteristics of the participants were surveyed with a questionnaire to measure depression, anxiety and stress levels using the Chinese Version of Depression Anxiety and Stress Scale-21 (DASS-21) (34, 35) at baseline and post-intervention. Previous studies have applied the Chinese version of DASS-21 to assess short and long-term psychological impacts of public health emergencies on people, such as SARS (36). In our study, the Chinese Version of DASS-21 was used to explore the participant' psychological health and to evaluate the effectiveness of CBT.

DASS-21 (37) is an abbreviated version of DASS-42. It contains three indicators, which are depression, anxiety and stress. Each indicator consists of 7 items, with a total of 21 items. Each item within the indicator was designed to measure the extent to which individuals have been bothered over past week, with a range of 0 to 3 (0 = “did not apply to me at all” to 3 = “apply to me very much, or most of the time”). Scores for depression, anxiety and stress were multiplied by two to calculate the final score according to the instructions of DASS-21 (37). Total scores of each indicator were ranged from 0 to 42, with higher scores indicating a higher-level psychological distress.

The depressive levels were defined as normal: 0–9, mild: 10–13, moderate: 14–20, severe: 21–27, and extremely severe: >27. Anxiety levels were defined as normal: 0–7, mild: 8–9, moderate: 10–14, severe: 15–19, and extremely severe: >19. Stress levels were defined as normal: 0-14, mild: 15–18, moderate: 19–25, severe: 26–33, and extremely severe >33. The DASS-21 has been widely established and used as reliable and valid assessment tool to access psychological health in adults in different countries (37–41).

The Chinese Version of DASS-21 developed by Taouk et al. in 2001 has been tested as a reliable and valid tool initially in Hong Kong, China. It was first introduced and applied to college students in mainland China in 2010 (42). In 2012, Wen et al. (35) slightly modified the Chinese Version of DASS-21 to make it more suitable for the Chinese culture and evaluated its reliability and validity in adults aged 18 years and above. The results indicated that the overall Cronbach's alpha coefficient of DASS-21 was 0.912 and the re-test reliability was 0.751, which showed that the Chinese Version of DASS-21 is a reliable and valid instrument and suitable for use in Chinese adults.

### Procedure

All participants were treated separately in a single isolation ward and were asked to complete the demographic characteristics questionnaire. The baseline assessment of depression, anxiety, and stress levels using the Chinese Version of DASS-21 were conducted within 24 h of hospital admission.

Participants in the control group received routine treatment (including antiviral treatment, symptomatic treatment of fever, and nursing care) according to the Chinese management guideline for COVID-19 (32). Participants in the intervention group received routine treatment with the addition of CBT including cognitive and behavior interventions.

The CBT has been described in detail in our previous study, mainly including cognitive intervention, relaxation techniques training, problem-solving training, and obtained a social support strategy for participants (43). The cognitive intervention aimed to help patients to correct their previous misconceptions in regard to COVID-19 information and management strategies. These included: (1) providing information that related to knowledge of COVID-19, real-time information of the COVID-19 outbreak such as number of patients who had been discharged or cured, and (2) giving clear and comprehensive explanations to patients' questions. The behavior intervention aimed to provide information on appropriate behaviors which help patients in coping with the COVID-19 pandemic. These included: (1) instruction on self-protection behaviors such as proper hand-washing technique, (2) self-monitoring COVID-19 related symptom such as fever and dry cough, (3) relaxation techniques such as music therapy and breath relaxation, (4) encourage patients to maintain close communication with family and friends through mobile phone or WeChat (a communication app). These information and intervention have been provided to patients in the intervention group. Participants were also asked to record their feelings and their medical adherence every day.

The CBT intervention was performed once a day in the morning, taking 30 min to complete and was recorded by the nurses. Each intervention was strictly carried out through face-to-face communication, with a patient centered approachh so the intervention could be adjusted based on the individual's needs. For example, the correct breathing relaxation methods, music choosing, and self-monitoring of chronic diseases strategy were instructed to meet personal preference. All the interveners strictly followed the required procedures according to the Technical Guidelines (44) to prevent them to be infected with COVID-19.

### Statistical Analysis

All data were entered at Excel then converted via Stat/Transfer to STATA/SE 14 (StataCorp, USA) for analysis. The level of statistically significance was set at *P* < 0.05.

Mean and standard deviation (SD) were presented for the continuous variables of age, length of hospital stays, scales of depression, anxiety, stress, and DASS-21 in the intervention and control groups at baseline. N (%) were presented for participants characteristics and categorial variables of different levels of depression, anxiety, stress, and DASS-21 in the intervention and control group at baseline. Analysis of variance (ANOVA) was used to compare the mean differences of continuous variables between intervention and control group at baseline. Chi-square tests were used to examine the statistical differences between intervention and control group by categorial variables at baseline.

The mean differences of continuous variables of DASS-21 indicators between baseline and post-intervention were examined by two-sided *t-*test for intervention and control group. The proportion differences of categorical variable of different levels DASS-21 indicators between baseline and post-intervention were examined by the proportion tests for intervention and control group.

The proportion differences between intervention and control group for each level of DASS-21 indicators were examined by proportion tests at baseline and post-intervention, respectively. We further calculated the changes of each DASS-21 indicator based on scores at two time-points, i.e., at baseline and post-intervention. Univariate linear regression was used to examine the association between chronic disease and changes of each DASS-21 indicator. Two-way scatter plots were generated to show the association of the length of hospital stay and the changes of each DASS-21 indicator by intervention and control groups.

## Results

Of a total of 93 participants, 35.5% were men and 64.5% were women. Average age was 48 years and mean length of hospital stay was 14.4 days. The majority of participants were married and had education level of secondary-graduate or below. Approximately half of participants were retired or unemployed, and 20.4% of them had chronic disease. At baseline, 50 (53.8%) participants had depression symptoms (mild: 34.4%, moderate: 18.3%, severe: 1.10% and extremely severe: 0%); 84 (90.3%) participants had anxiety symptoms (mild: 0%, moderate: 17.2%, severe: 40.8% and extremely severe: 32.3%) and 68 (73.1%) participants had stress symptoms (mild: 43.0%, moderate: 23.7%, severe: 6.40% and extremely severe: 0%).

Participants characteristics and each DASS-21 indicator (depression, anxiety and stress) by intervention and control group at baseline are shown in Table 1. There were no significant differences found between intervention and control group by participants characteristics and each DASS-21 indicator at baseline (*p* > 0.05).

**Table 1:** Participants characteristics and DASS-21 indicators by intervention and control group at baseline.

**Characteristics**	**Intervention group**	**Control group**	*P-*value*
	**(*N =* 47)**	**(*N =* 46)**	
**Age (years, SD)**	48.3 (12.2)	47.1 (10.6)	0.61
**Length of hospital stay (days, SD)**	14.3 (4.87)	14.3 (3.94)	0.92
**Gender (*****n*****, %)**			
Male	13 (27.7)	20 (43.5)	0.11
Female	34 (72.3)	26 (56.5)	
**Employment (*****n*****, %)**			
Employed	27 (57.5)	25 (54.4)	0.76
Unemployed/Retired	20 (43.5)	21 (45.7)	
**Education level (*****n*****, %)**			
Secondary and below	32 (68.1)	29 (63.0)	0.61
Tertiary	15 (31.9)	17 (37.0)	
**Marital Status (*****n*****, %)**			
Single	3 (6.38)	3 (6.52)	0.95
Married	39 (83.0)	39 (84.8)	
Divorce or others	5 (10.6)	4 (8.70)	
**Chronic disease status (*****n*****, %)**			
Have chronic disease	11 (23.4)	8 (17.4)	0.47
No chronic disease	36 (76.6)	38 (82.6)	
**Depression scale (mean, SD)**	11.0 (3.30)	10.1 (3.17)	0.15
**Anxiety scale (mean, SD)**	17.1 (4.44)	16.5 (4.81)	0.51
**Stress scale (mean, SD)**	16.8 (3.59)	17.1 (3.71)	0.74
**Total DASS-21 (mean, SD)**	45.0 (8.50)	43.7 (8.83)	0.46
**Depression level (*****n*****, %)**			
Normal	19 (40.4)	24 (52.2)	0.55
Mild	18 (38.3)	14 (30.4)	
Moderate	9 (19.2)	8 (17.4)	
Severe	1 (2.13)	0	
Extremely severe	0	0	
**Anxiety level (*****n*****, %)**			
Normal	4 (8.50)	5 (10.9)	0.35
Mild	0	0	
Moderate	5 (10.6)	11 (23.9)	
Severe	21 (44.7)	17 (37.0)	
Extremely severe	17 (36.2)	13 (28.3)	
**Stress level (*****n*****, %)**			
Normal	13 (27.7)	12 (26.1)	0.99
Mild	20 (43.6)	20 (43.5)	
Moderate	11 (23.4)	11 (23.9)	
Severe	3 (6.38)	3 (6.52)	
Extremely severe	0	0	

* *Analysis of variance (ANOVA) was used to compare the mean differences of age, length of hospital stay, scales of depression, anxiety, stress and DASS-21 between intervention and control group*.

*Chi-square test were used to compare the statistical differences between intervention and control group by participants characteristics, and the levels of depression, anxiety, stress, and DASS-21*.

Each DASS-21 indicator at baseline and post-intervention by intervention and control group are shown in Table 2. A significant decrease in means for scales of depression, anxiety, stress and total DASS-21 were found in both intervention (*p* < 0.001) and control groups (*p* = 0.001). Participants in the intervention group had a bigger deduction on means for scales of depression, anxiety, and total DASS-21.

**Table 2:** DASS-21 indicators at baseline and post-intervention by intervention and control group.

	**Baseline**	**After intervention**	**Mean/ percentage difference**	***P-*value**
	**Intervention group**			
Depression scale (mean, SD)*	11.0 (3.30)	7.98 (2.42)	−3.06 (3.68)	<0.001
Anxiety scale (mean, SD)*	17.1 (4.44)	10.3 (3.70)	−6.81 (5.16)	<0.001
Stress scale (mean, SD)*	16.8 (3.59)	13.1 (3.44)	−3.72 (3.52)	<0.001
Total DASS-21 (mean, SD)*	45.0 (8.50)	31.4 (7.42)	−13.6 (8.53)	<0.001
**Depression level (*****n*****, %)**^**#**^				
Normal	19 (40.4)	37 (78.7)	+18 (+38.3)	<0.001
Mild	18 (38.3)	9 (19.2)	−9 (−19.1)	0.04
Moderate	9 (19.2)	1 (2.13)	−8 (−17.1)	0.007
Severe	1 (2.13)	0	−1 (−2.13)	0.31
Extremely severe	0	0	0	–
**Anxiety level (*****n*****, %)**^**#**^				
Normal	4 (8.50)	22 (46.8)	+18 (+38.3)	<0.001
Mild	0	1 (2.13)	+1 (+2.13)	0.31
Moderate	5 (10.6)	17 (36.2)	+12 (+25.6)	0.004
Severe	21 (44.7)	7 (14.9)	−14 (−29.8)	0.002
Extremely severe	17 (36.2)	0	−17 (−36.2)	<0.001
**Stress level (*****n*****, %)**^**#**^				
Normal	13 (27.7)	29 (61.7)	+13 (+34.0)	<0.001
Mild	20 (43.6)	15 (31.9)	−4 (−11.7)	0.29
Moderate	11 (23.4)	2 (4.26)	−9 (−19.1)	0.007
Severe	3 (6.38)	1 (2.13)	−2 (−4.25)	0.31
Extremely severe	0	0	0	–
	**Control group**			
Depression scale (mean, SD)*	10.1 (3.17)	8.07 (2.06)	−2.00 (3.84)	0.001
Anxiety scale (mean, SD)*	16.5 (4.81)	11.2 (3.67)	−5.33 (4.91)	<0.001
Stress scale (mean, SD)*	17.1 (3.71)	12.8 (2.47)	−4.28 (4.20)	<0.001
Total DASS-21 (mean, SD)*	43.7 (8.83)	32.0 (6.45)	−11.6 (6.95)	<0.001
**Depression level (*****n*****, %)**^**#**^				
Normal	24 (52.2)	34 (73.9)	+10 (+21.7)	0.03
Mild	14 (30.4)	12 (26.1)	−2 (−4.3)	0.64
Moderate	8 (17.4)	0	−8 (−17.4)	0.003
Severe	0	0	0	–
Extremely severe	0	0	0	–
**Anxiety level (*****n*****, %)**^**#**^				
Normal	5 (10.9)	13 (28.3)	+8 (+17.4)	0.04
Mild	0	4 (8.70)	+4 (+8.70)	0.04
Moderate	11 (23.9)	21 (45.7)	+10 (+21.8)	0.04
Severe	17 (37.0)	8 (17.4)	−9 (−19.6)	0.03
Extremely severe	13 (28.3)	0	−13 (−28.3)	0.001
**Stress level (*****n*****, %)**^**#**^				
Normal	12 (26.1)	33 (71.7)	+11 (45.6)	<0.001
Mild	20 (43.5)	12 (26.1)	−8 (−17.4)	0.08
Moderate	11 (23.9)	1 (2.17)	−10 (−21.7)	0.002
Severe	3 (6.52)	0	−3 (−6.52)	0.08
Extremely severe	0	0	0	–

* *The mean differences between baseline and after intervention were examined by two-sided t-test*.

# *The proportion differences between baseline and after intervention were examined by proportion tests for intervention and control group*.

Significant increases in percentage of participants who had no depression symptom was observed in the intervention group (*p* < 0.001) and in the control group (*p* = 0.03). Significant decrease in percentage of participants who had mild (*p* = 0.04) and moderate (*p* = 0.007) depression symptom were observed in the intervention group, while only a significant decrease in percentage of participants who had moderate symptoms was found in the control group (*p* = 0.003).

In terms of anxiety, significant increases in the percentage of participants who had no anxiety symptom (*p* < 0.001) and had moderate anxiety symptoms (*p* = 0.004) were observed in the intervention group, whilst a significant increase in the percentage of participants who had no anxiety symptoms (*p* = 0.04), had mild (*p* = 0.04) and moderate (*p* = 0.04) anxiety symptoms were observed in the control group. A significant decrease in percentage of participants who had severe and extremely severe symptom was observed in both intervention and control groups (*p* < 0.05).

Significant increases in the percentage of participants who had no stress symptom was observed in both intervention and control groups (*p* < 0.001), whilst a significant decrease in the percentage of participants who had moderate stress symptoms was observed in both intervention and control groups (*p* = 0.007 and *p* = 0.002, respectively).

**Figure 2:**
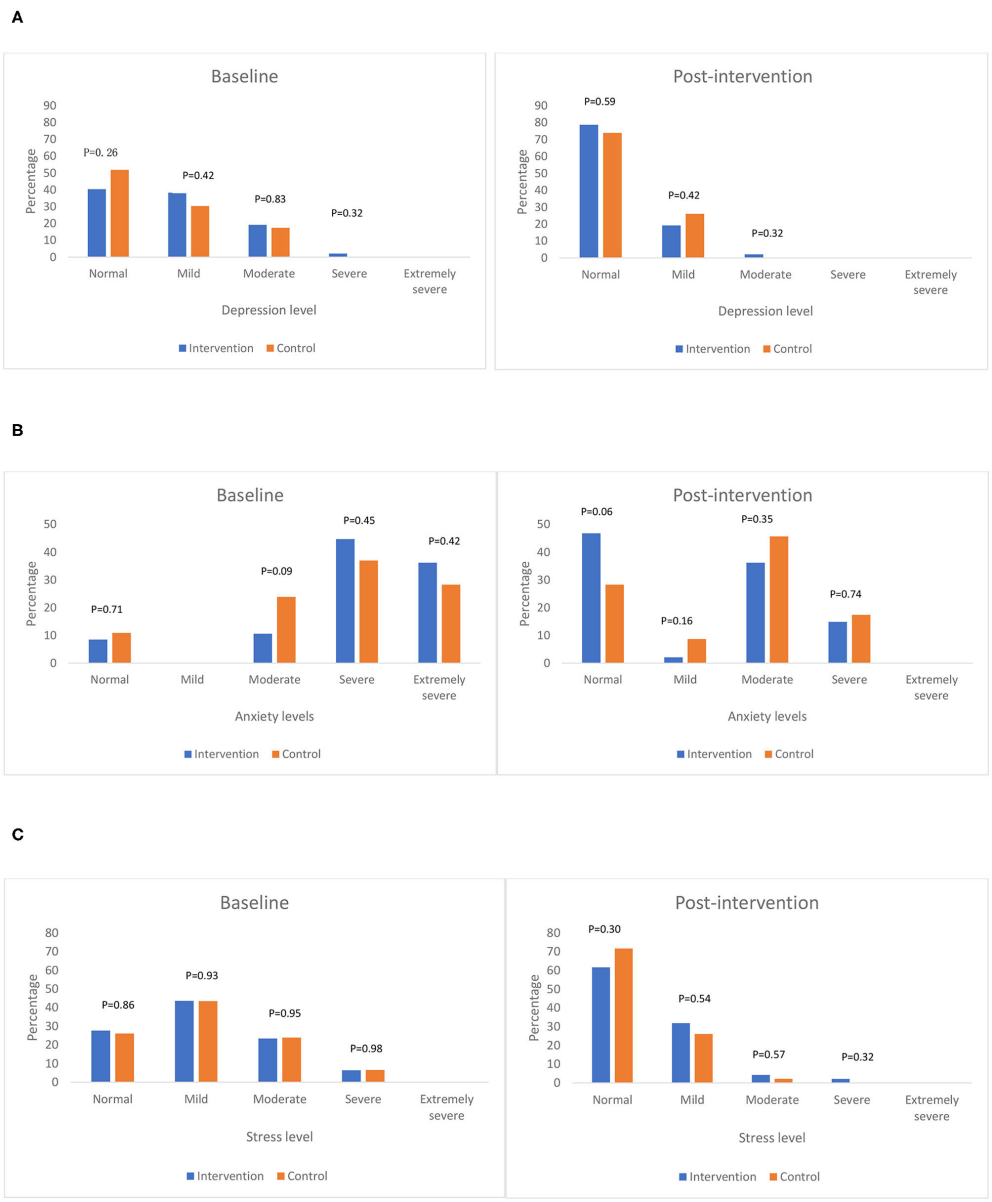
The percentage of each level of DASS-21 indicators by intervention and control group at baseline and post-intervention. **(A)** Depression level. **(B)** Anxiety level. **(C)** Stress level.

The percentage of each level of DASS-21 scale by intervention and control group at baseline and after intervention are shown in Figure 2. Overall, after intervention, all participants progressed toward a better depression, anxiety and stress status in both intervention and control groups. More participants had no depression and anxiety symptom in the intervention group than the control group, but no significant differences were found.

After intervention, less participants had moderate depression symptom, and no one had severe or extremely severe depression symptoms compared to baseline, meanwhile more participants have no depression symptoms in the intervention than the control group, but no statistical difference was observed (*p* = 0.59) (Figure 2A). Compared to baseline, less participants had severe anxiety symptoms, and no one had extremely severe anxiety symptoms after intervention. More participants have no anxiety symptoms in the intervention group than control group, but no statistical difference was observed (*p* = 0.06) (Figure 2B). After intervention, less participants had moderate and severe stress symptoms compared to baseline. Slightly less participants had no stress symptoms in the intervention group than control group, while a higher number of participants in the intervention than control group had mild stress symptoms. However, no statistical differences were observed (Figure 2C).

We further found significant associations between chronic disease status and changes in DASS-21 indicators (i.e., before and post-intervention) (Table 3). Compared with participants who had chronic disease, participants with no chronic disease had a bigger reduction across all DASS-21 indicators, with significant reductions found for total DASS-21 scale (coefficient = −4.74, 95% CI: −9.31; −0.17).

**Table 3:** The association between chronic disease status and changes of DASS-21 indicators.

**Chronic disease status**	**Coefficient (95% CI)**	***P-*value**
	**Changes of depression scale**	
Have chronic disease	0 (reference)	
No chronic disease	−1.87 (−3.77, 0.04)	0.05
	**Changes of anxiety scale**	
Have chronic disease	0 (reference)	
No chronic disease	−1.29 (−3.87, 1.30)	0.33
	**Changes of stress scale**	
Have chronic disease	0 (reference)	
No chronic disease	−1.59 (−3.54, 0.37)	0.11
	**Changes of DASS-21 scale changes**	
Have chronic disease	0 (reference)	
No chronic disease	−4.74 (−9.31, −0.17)	0.04

**Figure 3:**
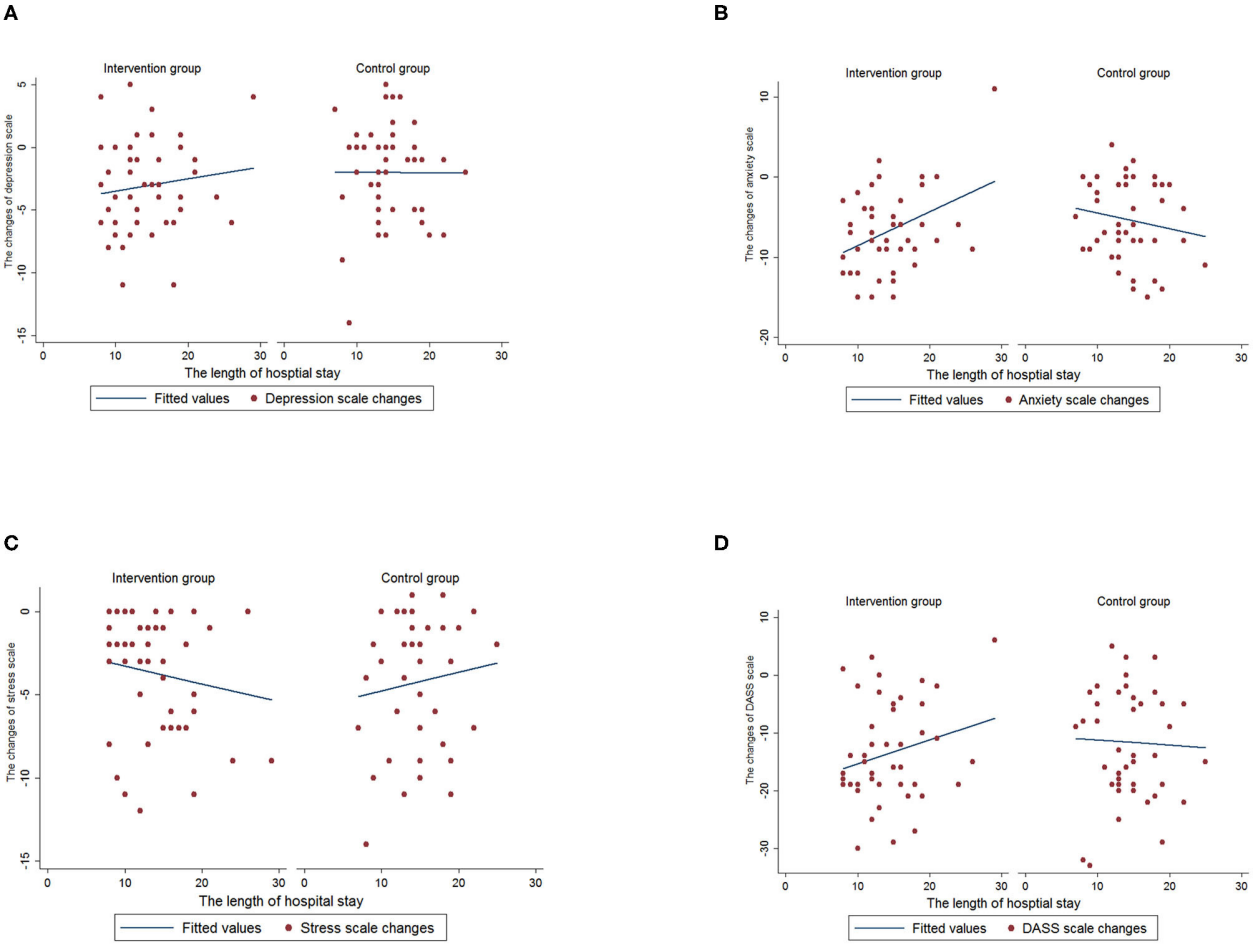
The association between the length of hospital stay and changes of DASS-21 indicators by intervention and control group. **(A)** Depression. **(B)** Anxiety. **(C)** Stress. **(D)** DASS-21.

The associations were also found between the length of hospital stay and changes of each DASS-21 indicator by intervention and control group (Figure 3). The length of hospital stay was significantly associated with a greater increase in anxiety scale in the intervention group (*p* = 0.005), but no significant association was found in the control group (*p* = 0.29). This indicated that participants who had a longer hospital stay were associated with less reduction of anxiety level after intervention. No significant associations were found between length of hospital stay and scales of depression (intervention group, *p* = 0.39; control group, *p* = 0.97), stress (intervention group, *p* = 0.32; control group, *p* = 0.49) and DASS-21 (intervention group, *p* = 0.11; control group, *p* = 0.81).

## Discussion

Our findings suggest that patients with COVID-19 experienced high levels of anxiety, depression and stress, which is consistent with previous studies (9–12, 17). To our best knowledge, our study is the first RCT to evaluate the effectiveness of CBT in reducing depression, anxiety and stress levels for patients with COVID-19 in China. Our results highlight the effectiveness of CBT intervention in improving psychological health for patients with COVID-19.

Our results indicate that the mean values of depression, anxiety, stress and total DASS-21 decreased significantly in both intervention and control groups after intervention. However, patients in the intervention group had a bigger mean deduction for scales of depression, anxiety and total DASS-21, indicating that CBT can effectively improve the psychological health of patients with COVID-19. These results are in line with previous studies (7, 45). Many studies have indicated that CBT is the most effective psychotherapy in reducing depression, anxiety and stress symptom that has been recommended as a first-line treatment for patients with psychological health disorders (31, 46, 47). Besides, the benefit of CBT is also shown in reducing insomnia and physical fatigue, which can further improve patients' quality of life (48, 49). Results from a number of systematic reviews and meta-analyses (50–52) have also found the effectiveness of CBT in improving short-term symptoms of depression, anxiety and related disorders and further reducing the risk of PTSD and social anxiety disorder.

Our results show more participants have no depression and anxiety symptoms in the intervention group than the control group although there were no statistical difference. These results are comparable to previous research findings. For example, Gromisch et al. (49) performed an RCT to evaluate the effectiveness of CBT in pain and depression and found that although there were no statistical significant difference between intervention and control groups for symptoms of pain and depression, an overall pain relief and improvement of depression symptom was observed in the intervention group.

As found in previous studies (7, 45, 53), we assumed that the main reason for the improvement of psychological health of patients after CBT intervention is because they received sufficient accurate information during the COVID-19 epidemic, along with timely clinical treatment, and were able to self-monitor their own health status. These all may contribute to the cognitive reconstruction process for patients, in which dysfunctional thinking patterns are constantly corrected, leading to the enhancement of self-confidence and self-efficacy, so as to reduce psychological suffering (54). Behavior interventions, including self-protection skills such as hand-washing technique, self-monitoring strategies and relaxation techniques such as music therapy, breathing relaxation, may also play an important role in effectively reducing patients' anxiety, depression and stress symptoms. Previous studies (55, 56) suggested that listening to pleasurable music can improve emotional self-regulation, executive function and cognition. Emotion processing within cortical and subcortical regions can be activated, which will increase the secretion of neurotransmitter dopamine, reduce the secretion of cortisol and further relieve stress and stress-related health issues (55–57). Studies have shown that breathing relaxation training leads to an overall reduction in sympathetic tone and an increase in parasympathetic output which combats increased sympathetic activities during stress (58, 59). This will contribute to a reduction of negative emotions such as stress, depression and anxiety (60–62).

We believe that patients who had close communication with family and friends and receive encouragement from medical staff helps them improve their psychological health. Previous studies have indicated that these strategies can enhance patient's self-confidence and reduce the psychological stress response caused by epidemics such as SARS and COVID-19. It can further have a great impact in promoting the physical and psychological health of patients (9, 53, 63, 64).

During the intervention, we found that by providing sufficient accurate information regarding COVID-19 management, patients' negative emotions have been significantly improved. In addition, we found that by improving of COVID-19 diagnosis, treatment procedure, (65), and implementing policies (e.g., free treatment for patients, timely nucleic acid testing and effective isolation and protection of family members), the psychological stress have been significantly relieved among patients with COVID-19.

Our results indicate that patients with chronic disease are not as able to improve their psychological health than patients without chronic disease. This implies that patients with chronic disease who receive CBT may not be able to significantly improve their psychological health in a relatively short time. In our study, 20.4% of patients with COVID-19 had a history of cardiovascular disease (CVD) or diabetes. Real-time data show severe COVID-19 disease and death are often associated with CVD and diabetes (14, 66). In addition, previous studies have widely reported that the symptoms of anxiety and depression in patients with CVD and diabetes are common and persistent (67, 68). This implies that the CBT intervention needs to be particularly focused in patients with chronic disease.

Our results also show that the patients with COVID-19 who have longer hospital stays are less likely improve their anxiety level in the intervention group. Patients who had longer hospital stay are patients with comorbidities requiring longer and more complex treatment. This can also lead to excessive worry and fear further aggravating psychological stress for patients and their family members. Therefore, our results find that CBT interventions need to be focused on patients who have a long duration of hospitalization.

## Strength and Limitations

To the best of our best knowledge, this is the first RCT study to evaluate the effectiveness of CBT in improving psychological health for patients with COVID-19 by using a rigorous study design and methodology, which is the main strength of this study. Secondly, all participants (except one patient who transferred to ICU) completed the entire study, which ensured data integrity. Thirdly, all health professionals and researchers who delivered the CBT received special professional training followed by a strict protocol, with the whole CBT intervention process guided and supervised by a psychological specialist. However, there are some limitations need to be recognized. A major limitation is the intervention period is relatively short, with a lack of long-term follow-up of participants after discharge. This may lead to a failure to fully understand and explain the impact of a CBT intervention. Secondly, there was a relatively small number of participants. However, during the COVID-19 outbreak, the traditional face-to-face CBT was almost impossible for a large number of patients due to the shortage of professionals and the rapid transmission of the virus.

## Conclusion

In conclusion, our study examined the effectiveness of CBT to improve psychological health including depression, anxiety, and stress in patients with COVID-19. Our results highlighted that CBT can effectively improve the psychological health of patients with COVID-19. Our results demonstrate that the CBT intervention needs to be particularly focused on patients with COVID-19 who have a chronic disease history and a longer hospitalization.

## Data Availability Statement

The datasets presented in this article are not readily available because it is stipulated in the informed consent signed with the patient that any information of the participants will not be disclosed, and the data collected for this study will be kept and stored by the first author. Requests to access the datasets should be directed to Jinzhi Li, 2639917769@qq.com.

## Ethics Statement

The studies involving human participants were reviewed and approved by Ethics Committee of the First Affiliated Hospital of Bengbu Medical College (approval number: BYYFY-2020KY10). The patients/participants provided their written informed consent to participate in this study.

## Author Contributions

JL proposed study topics, designed the study, and wrote the manuscript. XLi made contributions of designing the study and revised the manuscript. JJ, JW, YX, XuX, and XLin collected and verified the data. XiX made substantial contributions to the analysis, interpretation of data, edited, and revised the manuscript. JH edited and revised the manuscript. JX made contributions to the guidance of the study and revised the manuscript. HX participated in the design, and guidance of the study. All authors contributed to manuscript read and approved the submitted version.

## Conflict of Interest

The authors declare that the research was conducted in the absence of any commercial or financial relationships that could be construed as a potential conflict of interest.
